# Testosterone Degradative Pathway of *Novosphingobium tardaugens*

**DOI:** 10.3390/genes10110871

**Published:** 2019-10-31

**Authors:** Juan Ibero, Beatriz Galán, Eduardo Díaz, José L. García

**Affiliations:** Department of Microbial and Plant Biotechnology, Centro de Investigaciones Biológicas, Agencia Estatal Consejo Superior de Investigaciones Científicas. Ramiro de Maeztu 9, 28040 Madrid, Spain; jicaballero@cib.csic.es (J.I.); bgalan@cib.csic.es (B.G.); ediaz@cib.csic.es (E.D.)

**Keywords:** testosterone, steroid, catabolism, transcriptomic, biodegradation

## Abstract

In this work, we have shown that *Novosphingobium tardaugens* NBRC 16725 (strain ARI-1), a bacterial strain that was isolated due to its capacity to mineralize the estrogenic endocrine compound 17β-estradiol, is also able to mineralize testosterone, the androgenic endocrine compound. Using in silico analysis, we predicted a new putative steroid degradation (SD) gene cluster in strain ARI-1, which resembles genes involved in testosterone degradation in *Comamonas testosteroni* and other testosterone degrading bacteria like Actinobacteria (like *Rhodococcus* and *Mycobacteria* genera) although with significant differences in gene organization. A whole transcriptomic analysis of *N. tardaugens* revealed that testosterone produces a limited induction of the genes of the SD cluster that show a high basal expression in its absence. The 3β/17β-hydroxysteroid dehydrogenase involved in the first metabolic step of testosterone degradation was identified by using genetic and biochemical approaches. The construction of knockout mutant strains in the genes of the SD cluster together with in silico analyses suggests the existence of gene redundancy in the genome of *N. tardaugens.* This work will expand the knowledge about the metabolic pathways and biotransformation capabilities of a Gram-negative bacterium that could become a new model system in the bacterial steroid degradation field.

## 1. Introduction

Endocrine disruptors (EDCs) are chemicals that interfere with the endocrine system and produce adverse effects in both humans and wildlife. Numerous studies have reported the feminization and/or masculinization of freshwater wildlife exposed to estrogens and/or androgens in polluted rivers [1]. Natural estrogens and androgens enter the environment through the excretions of humans, domestic or farm animals, and wildlife. 17β-estradiol (E2) (estrogen) and testosterone (TES) (androgen), are the most ubiquitously sexual hormones found as pollutants in soil and water systems [2,3,4,5]. These compounds contaminate the waste water treatment plant effluents and occur at low concentration (ng/L to µg/L) [5,6]. Microbial degradation is a crucial mechanism to eliminate steroid hormones from contaminated systems and the persistence and fate of TES and E2 have been studied previously [3,5] showing that only 6% of E2 and 63% of TES could be mineralized in native soils under aerobic conditions. Similar results were obtained in water treatment plants [4]. This indicates that either a limited number of organisms can mineralize E2 [7,8,9,10,11,12,13,14] (Table S1) or that this compound is mineralized at lower rates compared to TES [15].

Although some bacteria are able to partially degrade/transform E2, only few of them, isolated mainly during the last 20 years, have been described to be able to completely metabolize E2 and use it as a sole carbon and energy source, either aerobically or anaerobically [15] (Table S1). In some cases, these bacteria need to grow in a rich medium to metabolize E2 (e.g., *Vibrio* sp. strain H5 [16] and *Buttiauxella* sp. strain S19-1 [17]). In other cases, a defined mixed culture of co-degraders are required to eliminate this compound (e.g., *Achromobacter xylosoxidans* and *Ralstonia* sp.) [8]. Nevertheless, there are only few bacteria described so far that can catabolize both E2 and TES [16,17,18,19,20,21,22,23] (Table S1).

*N. tardaugens* NBRC 16725 (strain ARI-1) is a Gram-negative, aerobic, rod-shaped and non-motile α-Proteobacterium isolated from a sewage treatment plant in Tokyo [7,24]. It was isolated due to its capacity to mineralize E2 and it has been used when immobilized in alginate to remove estrogens from sewage and cow dung [25]. Nevertheless, nobody has described so far its capacity to degrade TES or other androgens.

**Figure 1:**
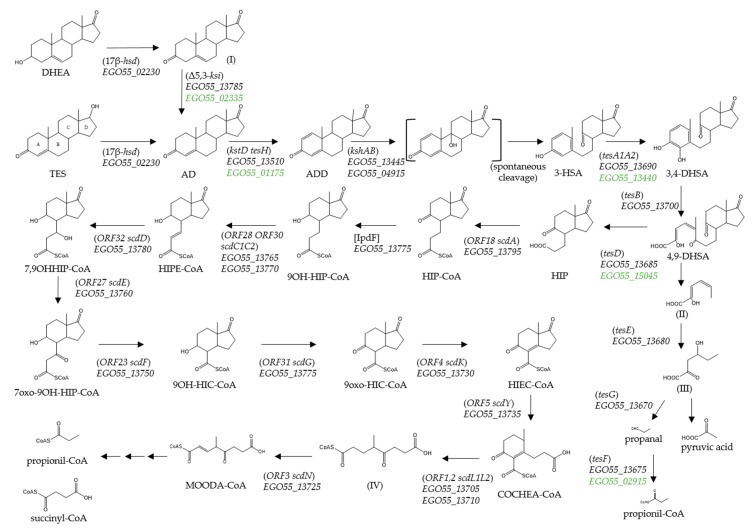
Proposed TES degradation pathway in *N. tardaugens*. Compound names are shown or indicated with an abbreviation: (I) androst-5-ene-3,17-dione; (3-HSA) 3-hydroxy-9,10-secoandrosta-1,3,5(10)-triene-9,17-dione; (3,4-DHSA) 3,4-dihydroxy-9,10-secoandrosta-1,3,5(10)-triene-9,17-dione; (4,9-DHSA) 4,5-9,10-diseco-3-hydroxy-5,9,17-trioxoandrosta-1(10),2-diene-4-oic acid; (II) 2-hydroxyhexa-2,4-dienoate; (III) 4-hydroxy-2-oxohexanoate; (HIP) 9,17-dioxo-1,2,3,4,10,19-hexanorandrostan-5-oic acid CoA ester; (HIPE-CoA) 9-hydroxy-17-oxo-1,2,3,4,10,19-hexanorandrost-6-en-5-oic acid; (9OH-HIC-CoA) 9α-hydroxy-17-oxo-1,2,3,4,5,6,10,19-octanorandrostan-7-oic acid, (HIEC-CoA) 9,17-dioxo-1,2,3,4,5,6,10,19-octanorandrost-8(14)-en-7-oic acid; (COCHEA-CoA) 9-oxo-1,2,3,4,5,6,10,19-octanor-13,17-secoandrost-8(14)-ene-7,17-dioic acid; (IV) 4-methyl-5-oxo-octane-1,8-dioic acid; and (MOODA-CoA) 4-methyl-5-oxo-oct-2-ene-1,8-dioic acid. Enzyme names are: (3,17β-*hsd*) 3,17β-hydroxysteroid dehydrogenase; (Δ5,3-*ksi*) 3-ketosteroid Δ4(5)-isomerase; (*kstD*) 3-ketosteroid-delta1-dehydrogenase; (*kshAB*) 3-ketosteroid 9alpha-hydroxylase; (*tesA1A2*) 3-HSA hydroxylase; (*tesB*) 3,4-DHSA 4,5-dioxygenase; (*tesE*) 2-hydroxyhexa-2,4-dienoate hydratase; (*tesG*) 4-hydroxy-2-oxovalerate aldolase; (*tesF*) propionaldehyde dehydrogenase; (*tesD*) 4,9-DHSA hydrolase; (*ORF18 scdA*) HIP-CoA ligase; [*IpdF*] 5-oxo HIC-CoA oxidase; (*ORF28 ORF30 scdC1C2*) acyl-CoA dehydrogenase; (*ORF32 scdD*) enoyl-CoA hydratase; (*ORF27 scdE*) dehydrogenase; (*ORF23 scdF*) CoA acetyl transferase; (*ORF31 scdG*) hydroxylacyl dehydrogenase; (*ORF4 scdK*) acyl-CoA dehydrogenase; (*ORF5 scdY*) enoyl-CoA hydratase; (*ORF1,2 scdL1L2*) β -ketoacyl-CoA-transferase; and (*ORF3 scdN*) CoA-hydratase. The proposed catabolic genes from *N. tardaugens* are indicated in italics by their locus code (*EGO55_XXXX*), in green other possible candidates are considered. The homologous genes from model bacteria *C. testosteroni* TA441 and *M. tuberculosis* H37Rv are shown in brackets and square brackets, respectively.

The complete oxic mineralization of TES has been studied in detail in *Comamonas testosteroni* [26,27] (Figure 1). The aerobic catabolism of TES is initiated by dehydrogenation of the 17β-hydroxyl group to produce androst-4-en-3,17-dione (AD), which undergoes a further dehydrogenation to form androsta-1,4-diene-3,17-dione (ADD). The subsequent cleavage of the core ring system is catalysed by several oxygenases that utilize oxygen as co-substrate [26,28] (Figure 1).

In this work, we have analysed the growth of *N. tardaugens* NBRC 16725 (strain ARI-1) with TES and other C-19 steroids as the sole carbon and energy sources. The complete genome sequence of this bacterium has been recently reported [29] allowing us to predict a SD gene cluster presumably involved in the catabolism of TES and other C-19 steroids. We have determined the expression of the catabolic genes by using a transcriptomic approach. The enzyme 17β-hydroxysteroid dehydrogenase, encoded outside of the predicted SD cluster, involved in the first step of TES catabolism in *N. tardaugens* ARI-1, has been identified by biochemical analyses and a metabolic pathway for the degradation of TES in this strain has been proposed.

## 2. Materials and Methods

### 2.1. Chemicals

Testosterone (TES), 17β-estradiol (E2), estrone (E1), pyruvate, chloroform, n-hexane, ethyl acetate, sulphuric acid and acetonitrile were purchased from Merck KGaA (Darmstadt, Germany). Androst-4-en-3,17-dione (AD) and androsta-1,4-diene-3,17-dione (ADD) were purchased from TCI EUROPE (Boereveldseweg, Belgium). TES-17-acetate (TES-Ac) was purchased from CYMIT QUÍMICA S.L. (Barcelona, Spain). Trans-dehydroandrosterone (DHEA) and pregnenolone (PREG) were purchased from Fluka (Switzerland). Randomly methylated β-cyclodextrin (TRMB-T Randomly Methylated BCD) (CDX) was purchased from Cyclodex (Alachua, USA). Other chemicals and reagents were purchased from Merck KGaA (Darmstadt, Germany).

### 2.2. Strains and Growth Media

Bacterial strains and plasmids used in this study are listed in Table S2. *N. tardaugens* NBRC 16725 (strain ARI-1) was purchased from the Leibniz-Institut DSMZ type culture collection. This strain and its mutants were cultured at 30 °C in an orbital shaker at 200 rpm. Nutrient broth (NB) (Difco) was used as rich medium to grow this strain. Minimal medium M63 [KH_2_PO_4_ (136 g/L), (NH_4_)_2_SO_4_ (20 g/L), FeSO_4_·7H_2_O (5 mg/L), pH 7.0] was supplemented with 0.39 mM CaCl_2_, 1 mM MgSO_4_ and the appropriate carbon source concentration (we used a carbon equimolar concentration for each substrate tested). Steroids and pyruvate stock solutions were prepared in PBS buffer and 70 mM CDX so the final carbon concentration in the culture was 36 mM in 13.33 mM CDX. *Escherichia coli* DH10B, BL21 (DE3) and HB101 strains were grown at 37 °C in an orbital shaker at 200 rpm in lysogeny broth (LB) medium [30]. The appropriate antibiotics, i.e., chloramphenicol (34 mg/mL), kanamycin (50 mg/mL) or rifampicin (50 mg/mL) were added when needed.

### 2.3. DNA Manipulation

Molecular biology and DNA manipulations where performed as described elsewhere [31]. *N. tardaugens* genomic DNA was extracted as described before [29]. Plasmid DNA was purified using High Pure Plasmid Isolation Kit (Roche). DNA fragments where purified with QIAquick PCR Purification Kit (Qiagen) or QIAquick Gel Extraction Kit (Qiagen). *E. coli* cells were transformed using the RbCl method or by electroporation using a Gene Pulser (Bio-Rad) [32]. DNA amplification was performed in a Mastercycler Gradient (Eppendorf) using the oligonucleotides listed in Table S3, which were purchased from Merck KGaA (Darmstadt, Germany). Phusion High-Fidelity DNA Polymerase (New England Biolabs) was used for cloning amplifications and Taq DNA polymerase (Biotools) for screening. All PCR products were checked by agarose gel electrophoresis and those aimed for cloning were confirmed by DNA sequencing by Secugen S.L. (Spain). Digestion of DNA fragments was done using restriction enzymes (New England Biolab) and ligation was performed with Instant Sticky-end Ligase Master Mix (New England Biolabs).

### 2.4. Gene Expression Analyses

#### 2.4.1. RNA Extraction

Total RNA of *N. tardaugens* cells was extracted from cultures grown in minimal medium with 20 mM CDX and TES or pyruvate as carbon sources. Cells where harvested in mid exponential phase (OD_600_ 0.6) and stored at −80 °C. Pellets where thawed and cells were lysed in 400 µL TE buffer (10 mM Tris-HCl, 1 mM EDTA, pH 7.5) containing lysozyme (50 mg/mL) following three freezing–thawing cycles. High Pure Isolation Kit (Roche), followed by DNA-free DNA Removal Kit (Invitrogen) treatment, was used to obtained pure RNA. Purity and concentration were measured in a ND1000 spectrophotometer (Nanodrop Technologies).

#### 2.4.2. RNA-seq

RNA-seq was done in Macrogen Korea. Total RNA integrity was checked using an Agilent Technologies 2100 Bioanalyzer. Ribosomal RNA was removed from the total RNA with Ribo-Zero rRNA Removal Kit to later construct a 100 bp paired-end library using TruSeq RNA Sample Prep Kit v2 that was quality-checked in an Agilent Technologies 2100 Bioanalyzer using a DNA 1000 chip. Library sequencing was performed in a HiSeq 3000 4000 (Illumina) using TruSeq 3000 4000 SBS Kit v3 as reagent. Bioinformatic analysis was performed by the Bioinformatics and Biostatistics service of the Centre for Biological Research (CIB-CSIC). Raw read data quality was checked using FastQC and trimmed with Trimmomatic. Trimmed reads were mapped against the genome sequence of *N. tardaugens* (accession number CP034179) using Bowtie2 and expression quantification was done using HTSeq-count. An average of 50 million raw sequencing reads (approximately 6.7 billion base pairs; average 1300× genome coverage per sample) were generated from samples from two independent experiments in the presence of pyruvate or TES, each with three biological replicates. After trimming the raw sequence reads, an average 48.7 million high-quality clean reads were mapped to the *N. tardaugens* reference genome and between 98.4% and 82.5% were uniquely mapped (Table S4). Differential expression analysis was done using Deseq2 and GO-term enrichment analysis was performed with GOSeq. The dissimilarity matrix shown in the heatmap was obtained with the euclidean distance and the cluster analysis was performed with the Ward’s minimum variance method. Bioinformatic analysis software was used with default settings. Raw read data obtained from the three replicates of the transcriptome of the strain grown in pyruvate and TES have been deposited in the Sequence Read Archive (SRA) database of the National Centre for Biotechnology Information (NCBI) under accession numbers SRR9027780, SRR9027781, SRR9027779 (Bioproject PRJNA541800) and SRR9027897, SRR9027898, SRR9027896 (Bioproject PRJNA541801), respectively.

### 2.5. Isolation of a Rifampicin Resistant Phenotype of N. tardaugens 

*N. tardaugens* wt strain, sensitive to rifampicin, was cultured in NB medium supplemented with rifampicin up to stationary phase (≈48 h). Cells where then plated on NB plates supplemented with the antibiotic and one single colony was picked, grown and used as *N. tardaugens* Rf^r^, suitable for conjugation.

### 2.6. Construction of N. tardaugens Knockout Strains

The knockout strains were constructed by double homologous recombination using the suicide vector pK18mobsacB [33]. *N. tardaugens* genomic DNA was used as template to amplify two fragments of ≈700 bp containing the upstream and downstream regions of the gene to delete. The fragments were digested with the appropriate restriction enzymes and cloned in the unique sites of the plasmid. The ligation product was transformed into *E. coli* DH10B competent cells and once recombinant candidates were PCR-checked, the cloned region was confirmed by sequencing. The plasmids were transformed by triparental conjugation [34] into *N. tardaugens* Rf^r^ as recipient strain using *E. coli* HB101 (pRK600) [35] as helper and *E. coli* DH10B, harbouring the corresponding vector, as donor. The strains resulting of plasmid integration in this first recombination event were selected in NB agar plates containing kanamycin and rifampicin and screened by PCR. Selected candidates were grown up to stationary phase (≈48 h) in NB medium and then plated in NB supplemented with 5% sucrose. The clones that are resistant to sucrose and sensitive to kanamycin were checked by PCR and the amplicon was sequenced to confirm the second cross-over event.

### 2.7. Heterologous Production of the Putative 3β/17β-Hydroxysteroid Dehydrogenase from N. tardaugens

To overproduce the putative 3β/17β-hydroxysteroid dehydrogenase (3β/17β-HSD), genes *EGO55_02235*-*EGO55_02230* (*hsd70-hsd60*) were cloned together or separately into the expression vector pET-29a. The DNA fragments containing *EGO55_02235-EGO55_02230, EGO55_02235* and *EGO55_02230* were amplified with primers 5′NdeIhsdTandemEcolif-3′BamHIhsdTandemEcolir, 5′NdeIhsd70SUBf-3′XhoIhsd70SUBr, 5′NdeIhsd60SUBf-3′XhoIhsd60SUBr, respectively (Table S3), using *N. tardaugens* genomic DNA as template. The DNA fragments were digested with the corresponding restriction enzymes *Nde*I-*Bam*HI, *Nde*I-*Xho*I and *Nde*I-*Xho*I respectively, and then ligated into the pET29a vector yielding pET29Hsd70-Hsd60, pET29Hsd70 and pET29Hsd60 plasmids, respectively. Electrocompetent cells of *E. coli* BL21 (DE3) were transformed with plasmids pET29Hsd70-Hsd60, pET29Hsd70 and pET29Hsd60. The resulting strains *E. coli* BL21 (DE3) (pET29Hsd70-Hsd60), *E. coli* BL21 (DE3) (pET29Hsd70) and *E. coli* BL21 (DE3) (pET29Hsd60) were grown in 50 mL LB containing kanamycin up to an OD_600_ of 0.5–0.8 and gene expression was induced with 0.2 mM IPTG (isopropyl-β-D-thiogalactopyranoside). After 3 h of induction, the cells were harvested, washed with 0.85% saline solution and resuspended in 20 mM Tris-HCl (pH 8.0). Cells were lysed by sonication using a Branson sonicator applying three cycles of 30-s bursts at maximum power alternated with 30 s cooling in ice. Soluble fraction was separated by centrifugation in a Sorvall Linx 6000 SS-34 rotor (15 min at 4 °C and 14000 rpm) and protein concentration was calculated using the Bradford method [36]. The overproduction of Hsd70 and Hsd60 proteins in the soluble fraction of the crude extract was checked by SDS-polyacrylamide gel electrophoresis (Figure S1).

### 2.8. Enzymatic Assay of 3β/17β-HSD Activity

Enzymatic assays of 3β/17β-HSD activity were performed in 500 µL total reaction volume. Reaction mixture consisted of 0.5 mg protein of the crude extracts obtained from the recombinant strains, 500 μM of the steroidal substrate dissolved in CDX (3.5 mM final concentration of CDX), 1 mM NAD+ and 50 mM sodium phosphate buffer (pH 7.0). Reaction assays were started by adding NAD+ and stopped by adding 2 volumes of chloroform.

### 2.9. Organic Phase Extraction and Thin Layer Chromatography (TLC) analysis

The presence of steroidal compounds in culture media and enzymatic assay mixtures was determined after organic solvent extraction by TLC analysis. Two volumes of chloroform were added and the mixture was vortexed for 30 s and centrifuged for 1 min at 13,000 rpm in an Eppendorf microcentrifuge. The organic phase was extracted and dried. The dried sample was dissolved in 100 μL of acetonitrile and analysed by thin layer chromatography (TLC). For TLC analysis, 10 µL of the standards and the samples dissolved in acetonitrile were spotted in silica gel plates (TLC Silicagel 60 F_254_, Merck Millipore) and n-hexane: ethyl acetate (10:4 v/v) was used as developing system. Steroid products were visualized by UV and revealed by spraying 20% (v/v) sulphuric acid and heating at 120 °C.

### 2.10. In Silico Analyses

Gene product prediction was done with Rapid Annotations using Subsystems Technology (RAST) [37]. Homologous genes search in different bacteria was performed by using the Standard Protein Basic Local Alignment Search Tool (BLASTp) [38].

## 3. Results

### 3.1. Catabolism of C-19 Compounds in N. tardaugens

**Figure 2:**
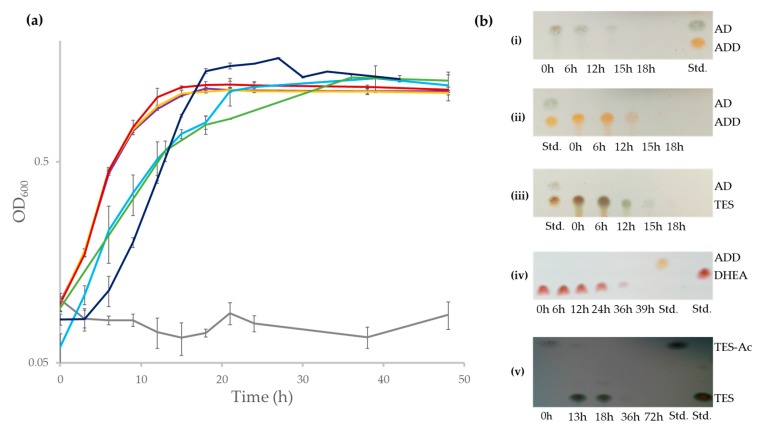
(**a**) Bacterial growth (Log_2_OD600) of *N. tardaugens* NBRC 16725 when cultured in M63 minimal medium containing 1.89 mM AD (purple), 1.89 mM ADD (yellow), 1.89 mM TES (red), 1.89 mM DHEA (light blue), 1.71 mM TES-Ac (green), 2 mM E2 (dark blue) and 13.33 mM CDX (grey) and (**b**) TLC analysis of the organic extraction of the culture of *N. tardaugens* along time growing in: (**i**) AD, (**ii**) ADD, (**iii**) TES, (**iv**) DHEA and (**v**) TES-Ac. The AD, ADD, TES, DHEA and TES-Ac standards (Std.) (1 mM) are also shown.

Although the ability of *N. tardaugens* strain ARI-1 to grow in E2 as a sole carbon and energy source has been described [24], nothing was known regarding its capacity to mineralize other C-19 steroids, such as TES, AD, ADD and DHEA. Figure 2a shows that *N. tardaugens* is able to mineralize these compounds. Moreover, we were unable to detect by TLC any of these steroids after 18 h cultivation (39 h in case of DHEA) (Figure 2b). Interestingly, strain ARI-1 was also able to grow in the xenobiotic steroid compound TES-Ac generating TES as transient intermediate (Figure 2a,b). Taken together all these results reveal that *N. tardaugens* contains an efficient aerobic degradative pathway for C-19 steroids.

### 3.2. In Silico Identification of N. tardaugens Genes for Catabolism of C-19 Compounds

**Figure 3:**
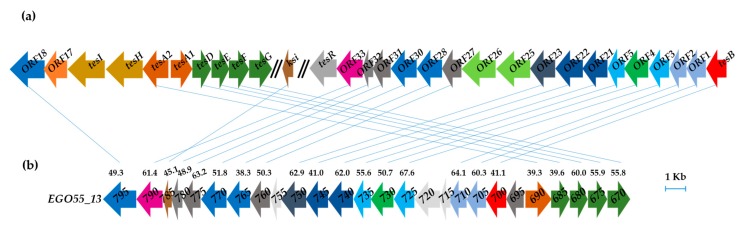
(**a**) *Comamonas testosteroni* TA441 mega-cluster of steroid degradation (accession number LC010134). (**b**) Putative steroid degradation gene cluster of *N. tardaugens* NBRC 16725 (accesion number CP034179). Genes encoding same funtion are pictured in the same color and connected with a line. Percent identities (BLASTp) of the gene products are pictured compared with those of *C. testosteroni*.

In silico analysis of the assembled *N. tardaugens* genome (4,358,096 bp) [29] using as query the amino acid sequences of the coding genes responsible for TES degradation in *C. testosteroni* TA441 revealed the existence of a putative steroid degradation gene cluster covering a 26.4 kb region (*EGO55_13795*-*EGO55_13670* (Figure 3). The predicted SD cluster is organized in two regions that are transcribed divergently, i.e., *EGO55_13690*-*EGO55_13670* and *EGO55_13695*-*EGO55_13795* (Figure 3b). In contrast to *C. testosteroni*, where the TES degradative genes are organized in two different clusters (Figure 3a), *N. tardaugens* shows a more compact gene organization.

Remarkably, the four genes essential for the three initial steps of C-19 steroid catabolism, i.e., the *hsd* gene encoding the 3β/17β-hydroxysteroid dehydrogenase (3β/17β-HSD), the *kstD* gene encoding the ketosteroid dehydrogenase, and the *kshA* and *kshB* genes encoding the oxidase and reductase subunits of the 9α-ketosteroid hydroxylase, respectively (Figure 1), are not contained within this degradation cluster. Interestingly, several genes homologous to *kstD*, *kshA* and *hsd* from *C. testosteroni* have been found distributed along the genome of *N. tardaugens* (Table S5). However, only one gene, *EGO55_04915*, encoded a protein showing identity (33%) with the reductase subunit of the ketosteroid hydroxylase *ORF17* (*kshB*) of *C. testosteroni*. Finally, a TeiR-like regulatory protein which is a positive regulator that induces the expression of the TES cluster in *C. testosteroni* [39], is not present in the *N. tardaugens* genome. Furthermore, the absence of other putative regulatory genes located near the SD cluster suggest either the absence of a specific regulation by TES or at least a different transcriptional regulation in this bacterium.

We have tried to demonstrate the involvement of the SD cluster in the degradation of C-19 compounds by constructing two *N. tardaugens* knockout mutant strains in two representative genes of the SD cluster, i.e., the *EGO55_13795* (*fadD3*) and *EGO55_13685* (*tesD*) genes. When both mutants were grown in TES as the sole carbon and energy sources their growth was not impaired (Figure S2). Interestingly, the analysis of the *N. tardaugens* genome revealed the existence of several homologous genes of the SD cluster located along the chromosome (Table S5) suggesting that some reactions involved in C19 steroid degradation could be replaced by such homologous genes. Thus, the analysis of the *N. tardaugens* genome revealed the existence of 9 *fadD3* and 3 *tesD* homologous genes (Table S5) that would explain the observed growth phenotype of the *fadD3* and *tesD* mutant strains.

### 3.3. Whole Transcriptomic Analysis of N. tardaugens Grown in Testosterone

**Figure 4:**
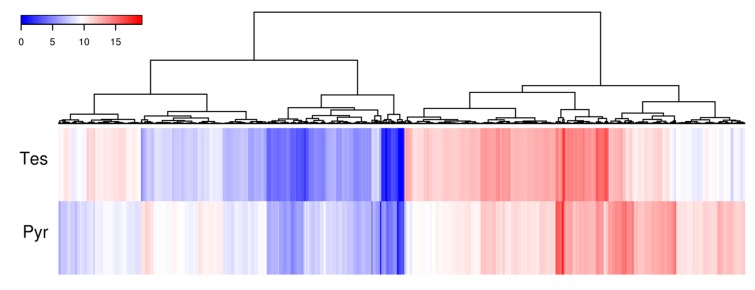
Heatmap diagram of cluster analysis showing the log2 mean normalize expression in each experimental growth condition for those genes where a FC > 2 and FC < −2 was observed.

**Figure 5:**
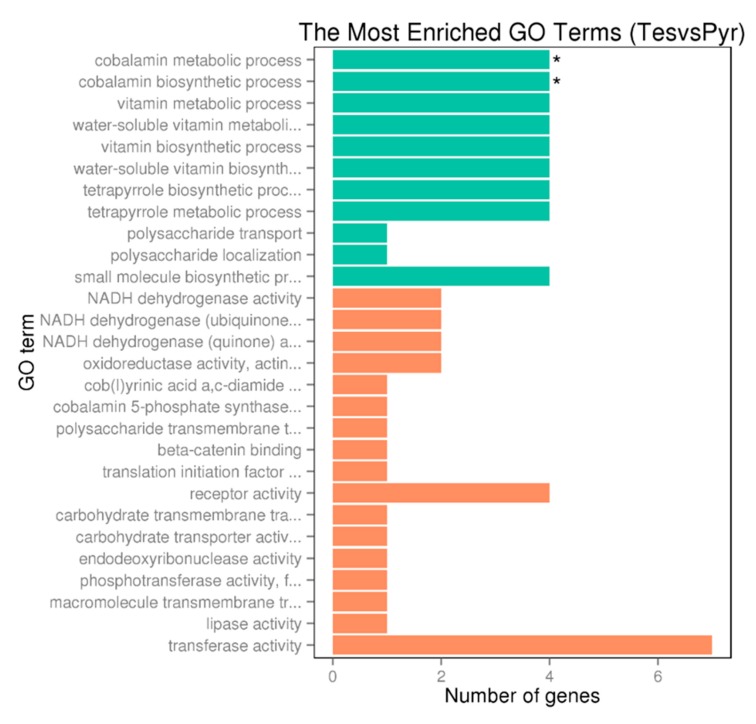
GO enrichment bar chart of upregulated genes (DEG) representing the number of DEGs enriched in biological process, cellular component and molecular function. Colors represent different GO types: biological process (green) and molecular function (orange). The term with a star “*” is significantly enriched term (corrected *p*_value_ < 0.05).

To determine the expression of the genes involved in the degradation of C-19 steroids we performed RNA-seq analyses in *N. tardaugens* cultured using pyruvate (control condition) or TES as carbon sources. Differential expression analysis yielded 2046 differentially expressed genes (DEGs) (from 3980 total genes in genome), where 863 were up regulated and 1183 were down regulated in TES condition compared to pyruvate (being FC 2 or −2, respectively, the cut-off value) (Table S6), showing a noticeable contrast in differential expression pattern (Figure 4). The functional classification of DEGs in different GO terms is shown in Figure 5. The highest level of up regulation (fold change (FC) > 10) was observed in 49 genes (Table S6) but other 111 genes were notably up regulated (5 < FC < 10) (Table S6).

The data obtained from the transcriptomic analysis show a slight differential induction of the SD cluster in the presence of TES with respect to the pyruvate control condition (Table S6). Nevertheless, it is important to notice that the basal expression level of these genes in pyruvate condition is already high when compared to that of housekeeping genes. The level of induction of the genes included in the *EGO55_13695*-*EGO55_13795* genes is slightly higher than those of the *EGO55_13690*-*EGO55_13670* genes (Table S6). Several genes showed a fourfold increase in expression levels: *EGO55_13735*, *EGO55_13740*, *EGO55_13745* and *EGO55_13750*. FC values of previously identified genes homologous to *kstD* and *kshA* from *C. testosteroni* TA441 (Table S5), allowed us to propose *EGO55_13510* (3.5-fold induction) and *EGO55_13445* (7.8-fold induction), as the KstD and KshA involved in TES degradation pathway in *N. tardaugens*, respectively (Figure 1). These *kstD* and *kshA* genes are located approximately 30 and 50 kb away from the SD cluster, respectively. Additionally, closed to the predicted kshA coding gene (*EGO55_13445*) there is a predicted TesA2 coding gene (*EGO55_13440*) that is induced 7.2-fold, which is a higher value than the one located within the SD cluster. Also the gene *EGO55_15045*, encoding a putative TesD homolog, showed higher induction than the corresponding gene in the SD cluster. These suggest that *EGO55_13440* and *EGO55_15045* genes could be involved in TES degradation in *N. tardaugens*.

Interestingly, *EGO55_01995*, *EGO55_02005*, *EGO55_02015* and *EGO55_02020* genes, encoding a putative propionyl-CoA carboxylase biotin-containing subunit, a methylmalonyl-CoA mutase, a methylmalonyl-CoA epimerase, and a propionyl-CoA carboxylase carboxyl transferase subunit, respectively, are found among the most upregulated genes in TES condition (Table S6). This result allowed us to identify a cluster (*EGO55_01990*-*EGO55_02025*) containing genes showing a high level of identity to those experimentally described as responsible of the methylmalonyl degradation pathway for propionic acid degradation [40,41,42,43,44] (Table S7). Furthermore, this cluster also contains a putative biotin synthase (*EGO55_02000*) that is upregulated, in agreement with the requirement of B7 cofactor for propionyl-CoA carboxylase activity [45] needed to metabolize propionyl-CoA, presumably generated in TES degradation (Figure 1).

As shown in Table S6, up to 19 genes annotated as involved in B12 cofactor biosynthesis pathway are significantly upregulated in TES condition. Figure 5 also shows that the highest number of upregulated genes in TES condition belong to cobalamin biosynthetic and metabolic processes, water-soluble vitamin biosynthesis, vitamin biosynthetic process and vitamin and water-soluble vitamin metabolic processes GO terms. High expression levels of genes involved in cobalamin synthesis pathway correlates with the requirement of this cofactor by the methylmalonyl-CoA mutase, which is upregulated in TES condition as indicated above (Table S6).

### 3.4. Identification of the Initial Biochemical Step of TES Degradation Pathway in N. tardaugens

In *C. testosteroni*, the degradation of TES starts by the dehydrogenation of the 17β-hydroxyl group to render AD. This step is catalysed by a short chain dehydrogenase 3β/17β-HSD, a tetrameric NAD(H)-dependent reversible enzyme [46]. Comparative gene analyses yielded up to 16 proteins homologous to the 3β/17β-HSD of *C. testosteroni* in *N. tardaugens* genome (Table S5). Due to this gene redundancy, we looked at the induction fold of those genes in the presence of TES. Among the 16 genes the *EGO55_02235* was slightly induced 2-fold and it is located in tandem with *EGO55_02230* encoding also a putative 3β/17β-HSD. These isoenzymes, Hsd60 and Hsd70, show 80% amino acid sequence identity. They form a putative four-gene operon together with the *EGO55_02225* gene coding for a putative esterase and the *EGO55_02240* gene annotated as a permease of the major facilitator superfamily. To prove the involvement of Hsd60 and Hsd70 in TES catabolism, a double knock out mutant was produced, where *EGO_02230* and *EGO_02235* genes were deleted. The mutant strain was grown in TES as sole carbon and energy source showing that its growth was not impaired (Figure S2). This result is not surprising given the number of homologous genes found in *N. tardaugens* genome (Table S5) that could be replacing the enzymatic activity of the deleted genes.

**Figure 6:**
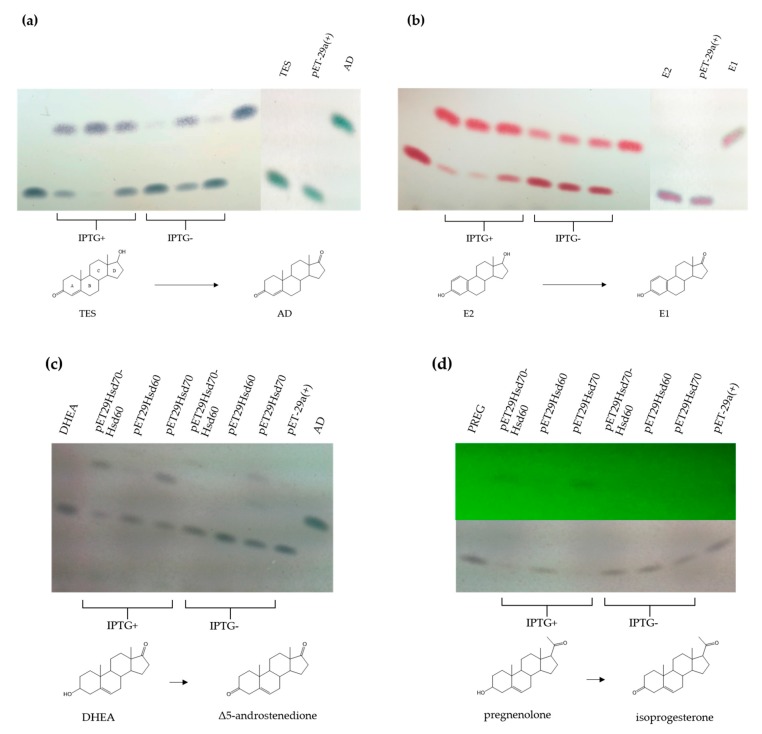
TLC analysis of the enzymatic reaction of crude extracts from *E. coli* BL21(DE3) cells harbouring pETHsd70-Hsd60, pETHsd70 and pETHsd60 transforming (**a**) TES, (**b**) E2, (**c**) DHEA and (**d**) PREG. In (**d**) the lower panel shows reduced products revealed with UV light. Standards of AD, TES, E2, E1, DHEA and PREG were also added. The use of inductor (IPTG) for overexpression is indicated. The molecular structure of the steroidal compounds involved in the reductive reactions are represented.

To further determine the putative role of Hsd60 and Hsd70 in TES metabolism the *EGO55_02230* and *EGO55_02235* genes were cloned in the pET29a vector and the resulting plasmid, named pET29Hsd70-Hsd60, was transformed in *E. coli* BL21 (DE3) cells to overproduce both enzymes (Figure S1). Enzymatic assays using crude extracts from *E. coli* BL21 (DE3) (pET29Hsd70-Hsd60) cells revealed a 17β-HSD reaction converting TES to AD (Figure 6a) and E2 to E1 (Figure 6b). Same enzymatic assays using DHEA and PREG as substrates showed that the catalytic activity present in the crude extract is able to transform them (according to their mobility in TLC) into the expected keto compounds, i.e., Δ5 androstadione and isoprogesterone, respectively (Figure 6c,d). The crude extract showed a lower activity when PREG (Figure 6d) was used as substrate, suggesting that the C-17 chain of PREG impairs the recognition of substrate. In this sense, no activity was detected when cholesterol with a long C-17 side chain was used as substrate.

Once the 3β/17β-HSD activity was determined within these cell extracts, the genes encoding Hsd70 and Hsd60 were cloned separately into the pET29a vector to explore their individual catalytic abilities. The resulting plasmids, pETHsd70 and pET29Hsd60, were transformed in *E. coli* BL21 (DE3) cells. Enzymatic assays using crude extracts showed, interestingly, that Hsd60 enzyme catalyzes more efficiently the dehydrogenation of C17-OH (Figure 6a,b), whereas Hsd70 catalyzes more efficiently the dehydrogenation of C3-OH (Figure 6c,d).

## 4. Discussion

*N. tardaugens*, ARI-1 strain was isolated based on its ability to metabolize E2 [24]. Here we show that in addition this strain has a prominent capability to metabolize other steroids. Particularly, we have demonstrated that this strain is able to degrade steroids that can also be considered as toxic EDCs, such as TES, TES-Ac, DHEA and other C-19 steroids, like AD and ADD (Figure 2).

The recent reduction of the number of contigs in a previous work [29] allowed us to identify the genes that could be responsible for the degradation of C-19 steroids in *N. tardaugens*. A comparison with the genes described for other C-19 degradation pathways, particularly with those described for the degradation of TES in *C. testosteroni*, was carried out. A significant finding was that ARI-1 contains most of the predicted genes in a very compact cluster compared with *C. testosteroni* that has at least two main clusters to encode the complete pathway (Figure 3) [26]. The same cluster structure as that of *N. tardaugens* has been described in other estrogen degrader strains such as *Sphingomonas* sp. KC8 strain and *Altererythrobacter estronivorus* [18]. In the particular case of the KC8 strain, those genes are being expressed in the E2 and TES grown cells, suggesting that strain KC8 uses the same gene products to degrade the C/D rings of both C18 and C19 compounds [18]. Since many of the genes of the SD cluster putatively responsible for CD-ring degradation of TES (C19) in *N. tardaugens* do not have any other homolog in the genome, it seems very likely that this cluster is also responsible for the degradation of C18 (E2) compounds. This is in accordance with recent work where a great level of conservation of predicted CD-ring degradation genes was found among different genera of steroid-metabolizing proteobacteria [47,48].

In *N. tardaugens*, only few genes required for C-19 degradation are located outside the SD cluster. We speculate that the genes *EGO55_02230* (*hsd*), *EGO55_13510* (*kstD*) or *EGO55_13445* (*kshA*), that are required to metabolize C19 compounds, but that are not included in the SD cluster, might be involved in the aerobic degradation of other steroid compounds, so its location outside the cluster suggests that they could be playing a more global role.

It is interesting that *kshB* (*EGO55_04915*) (*ORF17* in *C. testosteroni*) is not present in the SD cluster and no homologous gene to *tesA1* is found in the genome of *N. tardaugens*. KshB and TesA1 are the flavin reductase components of the flavin-dependent two-component monooxygenases KshA and TesA2, respectively. It has been demonstrated that these type of reductase components act in trans and are not highly specific and does not require a particular interaction [49,50]. However, it cannot be discarded that the reduced flavin required by KshA and TesA2 should be provided by unspecific reductases in *N. tardaugens*.

There are some genes included within the SD cluster of *N. tardaugens* that are not found in the TES cluster of *C. testosteroni*. This is the case of *EGO55_13755*, *EGO55_13720* and *EGO_13715*, which encode a nuclear transport factor 2 family protein, a lipid transfer protein and a benzoylsuccinyl-CoA thiolase, respectively. The precise role of these enzymes in the SD cluster remains unknown. On the contrary, there are some genes located within the TES cluster of *C. testosteroni* that have been located outside of the SD cluster. This is the case of *EGO55_13615* gene that is homologous to tesI gene from *C. testosteroni* encoding a ketosteroid Δ4-dehydrogenase, involved in epiandrosterone degradation [51]. In addition, the *ORF25* and *ORF26* genes of TES cluster encoding a 6-aminohexanoate-cyclic-dimer hydrolase are homologous to the *EGO55_02680* and *EGO55_04710* of *N. tardaugens*, respectively, that are located far from the SD cluster. These genes are also absent in the SD cluster of other estrogen-degrading strains like *Sphingomonas* sp. KC8 and *Altererythrobacter estronivorus* [18]. The role of these genes in TES catabolism in *C. testosteroni* is not clear since their disruption does not impair TES degradation [26].

Remarkably, we have detected a large number of genes dispersed in the genome of *N. tardaugens* that are homologous to those contained in the SD cluster and are expressed at significant levels in the presence of TES. This is the case of *EGO55_15045* (*tesD* homologous), *EGO55_02915* (*tesF* homologous), *EGO55_02335* (*ksi* homologous) and *EGO55_13440* (*tesA2* homologous), *EGO55_09255* and *EGO55_20150* (*ORF23* homologous), and *EGO55_01175* (*kstD* homologous) (Figure 1, Table S5). Therefore, we cannot discard the possibility that these genes could be involved in the metabolism of C-19 compounds in *N. tardaugens* enhancing the versatility and robustness of steroid metabolism.

In spite of the fact that the expression of other C-19 clusters, as occurs in *C. testosteroni*, are regulated by a specific regulator, in ARI-1 the data obtained from the transcriptomic analyses showed only a slight differential expression of the genes from the SD cluster in the presence of TES (Table S6). The catabolic enzymes for TES degradation described so far were not constitutively expressed but, rather, were significantly induced by their respective substrates [39,52]. For instance, in *C. testosteroni* the LuxR-type transcription activator TeiR regulates the transcription of genes involved in the initial enzymatic steps of TES degradation [39]. Moreover, the *teiR* deletion mutant was not able to use TES as a carbon source. TesR from *C. testosteroni* TA441, almost identical to that of TeiR from *C. testosteroni* ATCC11996, was shown to be necessary for induction of the steroid degradation gene clusters, *tesB* to *tesR*, *tesA1* to *tesG*, and *tesA2* to *ORF18* in *C. testosteroni* TA441 [53]. Interestingly, it has been reported that *tesR*-like regulatory genes are only present in *C. testosteroni* strains but are not found in other testosterone-degrading bacteria [26]. The in silico analysis showed that neither a *teiR* homologue nor other putative regulatory genes are present in the vicinity of the SD cluster in ARI-1. This observation is consistent with the transcriptomic analysis revealing a significant basal expression of the SD cluster in the absence of TES, which might be explained because this pathway could be fundamental for the survival of the ARI-1 strain in the specific niche where it was isolated.

There are other genes that appear to be induced by pleiotropic induction effects in the presence of TES and which are not directly related to the degradative C-19 pathway but rather to central metabolic processes (e.g., stress processes, requirements of cofactor synthesis, etc.). For instance, the production of propionyl-CoA as a presumable metabolite of TES degradation or the requirements of CoA to mineralize the TES rings can promote the generation of multiple stresses and pleiotropic differential expression signals when compared with the transcriptome of pyruvate metabolism [54,55]. According to the metabolic scheme shown in Figure 1, TES would be converted into succinyl-CoA (1 mol), pyruvate (1 mol), acetyl-CoA (3 mol) and propionyl-CoA (2 mol). The analysis of the genome revealed that *N. tardaugens* does not have a methylcitrate cycle and thus, it metabolizes propionyl-CoA by the methylmalonyl-CoA pathway to synthesize succinyl-CoA. The finding that the methyl-malonyl-CoA pathway is highly upregulated under TES growth conditions, is a solid evidence that propionyl-CoA is formed.

3β/17β-HSDs are essential enzymes in the biosynthesis of all classes of mammalian steroids. They catalyze the interconversion of alcohol and carbonyl functions stereospecifically in defined positions using oxidized or reduced NAD(H) or NADP(H) as co-substrates [56]. It is also well known that 3β/17β-HSDs are involved in the first catabolic step in TES degradation and, particularly, the 3β/17β-HSD of *C. testosteroni* has been extensively studied [20,46,57,58]. It catalyzes the reversible reduction/dehydrogenation of the oxo/β-hydroxy groups at positions C3 and C17 of steroids, including hormones and isobile acids. The dual positional specificity of this 3β/17β-HSD has been explained after resolving its 3D structure [46]. Kinetic studies revealed an ordered mechanism and suggested a single catalytic site accommodating both the 3β and 17β activities [46]. *N. tardaugens* has 16 genes that might encode putative 3β/17β-HSD homologous to the enzyme described in *C. testosteroni* (Table S5). The transcriptomic data obtained in the presence of TES allowed us to postulate specific candidates that could be involved in the metabolism of TES. Furthermore, after exploring the genetic environment of the 16 candidates, we identified the *EGO55_02235*-*EGO55_02230* tandem genes that code for two putative 3β/17β-HSD isoenzymes (Hsd60 and Hsd70) as part of a putative four-genes operon together with the *EGO55_02225* gene coding for a putative esterase and the *EGO55_02240* gene encoding a putative permease of the major facilitator superfamily. Taking into account all these reasons we cloned and tested the activity of the two putative HSDs of this operon demonstrating that they have a differential activity on C-19 steroids (Figure 6). Our results showed that isoenzyme Hsd60 is more efficient performing the C17-OH dehydrogenation than Hsd70 (Figure 6a,b), which in turn performed more efficiently the dehydrogenation of C3-OH (Figure 6c,d). The substrate specificity of Hsd60 makes this enzyme a very interesting candidate for the development of biocatalysts for TES production using mycobacterial strains that accumulate AD as described [59]. The alignment of the two proteins showed a variable region at the carboxyl-terminal domain (196*–*230 residues) that only share 42% amino acid sequence identity, in contrast with the 80% of the whole polypeptide chain. It has been described that the steroid binding pocket in 3α, 20β-HSD from Streptomyces hydrogenans is formed by the carboxyl-terminal 60 residues [60]. Interestingly, the sequence alignment of 3α, 20β-HSD with Hsd60 and Hsd70 showed that the variable region detected for both isoenzymes align perfectly with the sequence responsible for steroid binding at 3α, 20β-HSD (Figure S3). The lower similarity of this region is consistent with the variable substrate specificity observed between the two isoenzymes. 

It is well known that many steroids are currently used as pharmaceutical drugs in their esterified forms. The esterification of natural or synthetic androgens or anabolic steroids renders metabolic resistant prohormones with improved oral bioavailability, increased lipophilicity, and extended elimination half-life. This is for instance the case of TES that can be esterified with acetate (TES-Ac) that was first described in 1936 and was one of the first androgen esters to be synthesized and used as an anabolic steroid. To introduce steroid esters in the C-19 degradation pathway they should be firstly hydrolyzed by an esterase. On the other hand, the uptake of these lipophilic xenobiotic compounds might require a specific transport system to cross the two membranes of a Gram-negative bacterium. Then, the production of an esterase together with two HSD enzymes and the assistance of a transport protein appear to constitute an efficient system to handle the degradation of these pharmaceutical compounds that frequently contaminate the municipal waste waters from where *N. tardaugens* was isolated [7]. In agreement with this hypothesis, we have demonstrated that *N. tardaugens* is able to catabolize TES-Ac (Figure 2).

The fact that *N. tardaugens* can degrade both C3-OH and C17-OH steroids (Figure 2) supports the existence of both HSD activities. This finding does not rule out that some of the other HSD homologous enzymes encoded in the genome of *N. tardaugens* can fulfil these activities and additional experiments should be carried out to demonstrate a specific implication of this operon in the metabolism of steroids. Nevertheless, this result opens a new and interesting scenario to study in more detail the degradation of xenobiotic steroid esters of EDCs.

Several examples of bacteria capable of using both cholesterol and C-19 steroids as the only carbon and energy source have been described (e.g., *Rhodococcus erythropolis* strain SQ1 [61], *Rhodococcus ruber* strain Chol-4 [62], *Rhodococcus rhodochrous* DSM 43269 [63], *Gordonia neofelifaecis* NRRL B-59395 [64]). Using the genes of the SD cluster of *N. tardaugens* as a template, we screened the presence of homologous genes in other steroid metabolizing bacteria (Table S8). Apart from *C. testosteroni* TA441, we observed homologous genes in *Sphingomonas* sp. KC8, *Pseudomonas* sp. Chol1, *Rhodococcus jostii* RHA1, *Mycobacterium tuberculosis* H37Rv, *Mycobacterium smegmatis* mc^2^155, *Altererythrobacter estronivorus* MH-B5 and *Sterolibacterium denitrificans* Chol-1S(T) genomes. In spite of their large phylogenetic distances, a significant identity was observed with some Actinobacteria, e.g., RHA1, H37Rv and mc^2^155 strains (Table S8). This suggests a great level of conservation of key degradative enzymes among bacteria adapted to metabolize different steroidal compound.

Genetic manipulation of the ARI-1 strain will pave the way to unravel in more detail not only TES degradative pathway but also the other pathways involved in the degradation of E2 and several steroids that are used as carbon and energy sources by this bacterium. The data presented will enable to build upon the knowledge on metabolic pathways and the biotransformation capabilities of this Gram-negative bacterium that could become a new, model system in the steroid field. Moreover, *N. tardaugens* NBRC 16725 (strain ARI-1) might cover a wide spectrum of steroid biotransformation reactions and their improvement may lead to promising alternative biotechnological processes.

## Supplementary Materials

The following are available online at https://www.mdpi.com/2073-4425/10/11/871/s1, Figure S1: 12.5% SDS-polyacrylamide gel electrophoresis of the overproduction of Hsd70-Hsd60, Hsd60 and Hsd70 proteins in the soluble fraction of the crude extract of *E. coli* BL21(DE3) strains.Twenty-five µg of total protein of each sample where loaded, Figure S2: Bacterial growth (Log_2_OD_600_) of *N. tardaugens* NBRC 16725 (red), *N. tardaugens* Δ*fadD3* (yellow), *N. tardaugens* Δ*tesD* (purple) and *N. tardaugens* Δ*hsd* (green) strains when cultured in M63 minimal medium containing 1.89 mM TES, Figure S3: Protein alignment of the 3α,20β-HSD from *Streptomycens hydrogenans* with HSD60 and HSD70. The key amino acids of the active site are in red. The variable sequence is shown in a red box. This sequence is involved in substrate binding in the 3α, 20β-HSD from *S. hydrogenans*, Table S1: Isolated bacteria able to degrade E2 and/or TES, Table S2: Bacterial strains and plasmids used in this study, Table S3: Primers used in this study, Table S4: Statistical results of whole genome sequencing and mapping of all transcripts, Table S5: Genes found in *N. tardaugens* genome homologous to genes involved in testosterone degradation in *C. testosteroni* TA441. Percentage of identity (ID %) and fold change (FC) increase in expression levels when *N. tardaugens* grows in testosterone are shown, Table S6: Gene expression analysis (RNA-seq) of *N. tardaugens* grown in testosterone condition compared to pyruvate. Genes located in the SD cluster (blue) and those involved in methylmalonyl-CoA pathway (green) and cofactor B12 biosynthesis pathway (orange) are highlighted, Table S7: Methylmalonyl-CoA degradation cluster in *N. tardaugens* NBRC 16725. Genes homologous to those described as involved in the pathway are highlighted in green, Table S8: Steroid degradation genes in the putative testosterone degradation pathway of *N. tardaugens* (accession CP034179). Homologous genes found in the genomes of *C. testosteroni* TA441 (accession LC010134), *Sphingomonas* sp. KC8 (accession CP016306), *Pseudomonas* sp. Chol1 (accession AMSL00000000), *R. jostii* RHA1 (accession CP000431), *M. tuberculosis* H37Rv (accession AL123456.3), *M. smegmatis* mc2 155 (accession CP000480), *A. estronivorus* MH-B5 (accession NZ_JRQQ00000000) and *S. denitrificans* Chol (accession LT837803) are listed and the percentage identity is shown. A cut-off value of 39 % identity was used.
