# Supplementary material for: Testosterone Degradative Pathway of Novosphingobium tardaugens
Source: Genes (Basel). 2019 Oct 31;10(11):871. doi: 10.3390/genes10110871 (PMC6895838; doi:10.3390/genes10110871)
Supplement: Supplementary file 1 [file genes-10-00871-s001.zip › genes-515246 supplementary/genes-599144-supplementary.docx]

**Table S1.** Isolated bacteria able to degrade E2 and/or TES.

| **Bacterial strain** | **Compound** | **Condition** | **Reference** |
| --- | --- | --- | --- |
| *Alcaligenes* sp. (strain M21) | E2, TES | Aerobic | [20] |
| *Novosphingobium tardaugens* NBRC 16725 (strain ARI-1) | E2 | Aerobic | [7] |
| *Sphingomonas* sp. (strain KC8) | E2, TES | Aerobic | [18] |
| *Novosphingobium* sp. (strain E2S) | E2, TES | Aerobic | [19] |
| *Sphingobacterium* sp. (strain JCR5) | E2 | Aerobic | [9] |
| *Bacillus* spp. | E2 | Aerobic | [10] |
| *Sphingomonas* sp. (strain ED8) | E2 | Aerobic | [11] |
| *Pseudomonas aeruginosa* (strain TJ1) | E2 | Aerobic | [12] |
| *Novosphingobium* sp. (strain JEM-1) | E2, TES | Aerobic | [21] |
| *Rhodococcus zopfii* (strain Y50158) | E2, TES | Aerobic | [22] |
| *Rhodococcus equi* | E2, TES | Aerobic | [22] |
| *Novosphingobium* sp. (strain SLCC) | E2 | Aerobic | [13] |
| *Vibrio* sp. (strain H5) | E2, TES | Aerobic | [16] |
| *Buttiauxella* sp. (strain S19-1) | E2, TES | Aerobic | [17] |
| *Achromobacter xylosoxidans + Ralstonia* sp. | E2 | Aerobic | [8] |
| *Steroidobacter denitrificans* (strain FST) | E2, TES | Anaerobic | [23] |
| *Denitratisoma oestradiolicum* (DSM 16959(T); strain AcBE2-1(T)) | E2 | Anaerobic | [14] |

**Table S2.** Bacterial strains and plasmids used in this study.

| **Strains and plasmids** |  |  |
| --- | --- | --- |
| **Strains** | **Genotype and characteristics** | **Source/reference** |
| *N. tardaugens* NBRC 16725 | wild type strain | [7] |
| *N. tardaugens* Rf^r^ | Rf^r^ strain efficient for conjugation | This study |
| *N. tardaugens* NBRC 16725 Δ*fadD3* | *N. tardaugens* NBRC 16725 Δ*EGO55_13795* | This study |
| *N. tardaugens* NBRC 16725 Δ*tesD* | *N. tardaugens* NBRC 16725 Δ*EGO55_13685* | This study |
| *N. tardaugens* NBRC 16725 Δ*hsd* | *N. tardaugens* NBRC 16725 Δ*EGO55_02230-EGO_02235* | This study |
| *E. coli* DH10B | F^-^, mcrA, Δ(*mrr hsdRMS*-*mcrBC*), Φ80d*lacZ*ΔM15, Δ*lacX74*, *deoR*, *recA1*, *araD139*, Δ(*ara-leu*)7697, *galU*, *galK*, λ^-^, *rpsL*, *endA1*, *nupG* | Invitrogen |
| *E. coli* BL21 (DE3) | F^-^, *ompT*, *hsdSB*(r^-^B m^-^B ) *gal dcm* λDE3 (harbouring gene *1* of the RNA polymerase from phage T7 under the *PlacUV5* promoter) | [31] |
| *E. coli* HB101 | *hsdS20* (r_B_^-^m_B_^-^) *recA13* *ara-14* *lacY1 galK2 rpsL20* (Sm^r^) *xyl-5 mtl-1 supE44* | [31] |
|  |  |  |
| **Plasmids** |  |  |
| pRK600 | Cm^r^ ColE1*oriV* RP4 *oriT*; helper plasmid in triparental matings | [35] |
| pK18*mob*sacB | Km^r^, ColE *oriV*, Mob+, *lacZα*, *sacB*; vector for allelic exchange homologous recombination mutagenesis | [33] |
| pK18*mob*sacB-fadD3 | pK18*mob*sacB derivative used for *EGO55_13795* deletion | This study |
| pK18*mob*sacB-tesD | pK18*mob*sacB derivative used for *EGO55_13685* deletion | This study |
| pK18*mob*sacB-HSD | pK18*mob*sacB derivative used for *EGO_02230-EGO55_02235* deletion | This study |
| pET-29a(+) | Cloning and expression vector, Km^r^, *ori*ColE1, *T7* promoter | Novagen |
| pET29Hsd60 | pET-29 containing *EGO55_02230* gene | This study |
| pET29Hsd70 | pET-29 containing *EGO55_02235* gene | This study |
| pET29Hsd70-Hsd60 | pET-29a(+) containing *EGO55_02235*-*EGO55_02230* tandem | This study |

**Table S3.** Primers used in this study.

| **Primers** | **Sequence (5'-3')** | **Use** |
| --- | --- | --- |
| 5’NdeIhsdTandemEcolif | CACACCATATGATGGGCCGACTTTCCG | Cloning tandem *EGO55_02235*- *EGO55_02230* into pET29a(+) |
| 3’ BamHIhsdTandemEcolir | CAGGGATCCTCATGCCTCCGGCACG | Cloning tandem *EGO55_02235*- *EGO55_02230* into pET29a(+) |
| 5'NdeIhsd70SUBf | ACAGCATATGATGGGCCGACTTTCCGGC | Cloning *EGO55_02235* into pET29a(+) |
| 3'XhoIhsd70SUBr | CATACCTCGAGTCAGGCTTCGGGCACACC | Cloning *EGO55_02235* into pET29a(+) |
| 5'NdeIhsd60SUBf | ACAGCATATGATGGGTCGGCTTGCTGGC | Cloning *EGO55_02230* into pET29a(+) |
| 3'XhoIhsd60SUBr | CATACCTCGAGTCATGCCTCCGGCACGCC | Cloning *EGO55_02230* into pET29a(+) |
| 5BamHIFadD3UPf | CATACGGATCCGGACCAACAAGCGGACCTAT | *EGO55_13795* deletion (amplification of upstream region) |
| 3SalIFadD3UPr | CACAGTCGACTCTCGTCGCATGCAGAAACC | *EGO55_13795* deletion (amplification of upstream region) |
| 5SalIFadD3DOWNf | CACAGTCGACTGTCAGGCGACTTCGAACAG | *EGO55_13795* deletion (amplification of downstream region) |
| 3HindIIIFadD3DOWNr | ACATAAGCTTTGAGGCGCAGGATCGTTTC | *EGO55_13795* deletion (amplification of downstream region) |
| 5TesDUPEcoRIf | ATACTGAATTCGGCAGCCATGACGTGATTG | *EGO55_13685* deletion (amplification of upstream region) |
| 3TesDUPBamHIr | CACATGGATCCTGGATGAATACCACGGCAGG | *EGO55_13685* deletion (amplification of upstream region) |
| 5TesDDOWNBamHIf | ATACTGGATCCCGTCCGCGCAATCACATT | *EGO55_13685* deletion (amplification of downstream region) |
| 3TesDDOWNHindIIIr | CACATAAGCTTCCAGCGTGTTGGCCAGC | *EGO55_13685* deletion (amplification of downstream region) |
| 5HSDUPEcoRIf | CCGGAATTCGTGTCTTGAGAACCAGCCCC | *EGO55_02235*-*EGO55_02230* deletion (amplification of upstream region) |
| 3HSDUPBamHIr | CACGGATCCTGGCGGGCACATACTATCAG | *EGO55_02235*-*EGO55_02230* deletion (amplification of upstream region) |
| 5HSDDOWNBamHIf | CAAGGATCCCACGTCGGTGATCGCTAC | *EGO55_02235*-*EGO55_02230* deletion (amplification of downstream region) |
| 3HSDDOWNHindIIIr | CCCAAGCTTTCGTGTCCTTTGCCATCG | *EGO55_02235*-*EGO55_02230* deletion (amplification of downstream region) |
| extFadD3f | AGGATACCGGCAAACACAGC | External primer: together with F24 used to check insertion of pK18mobsacB-fadD3. Together with extFadD3r used to check *EGO55_13795* deletion |
| extFadD3r | TGGTCATGATCGAAACCGCT | External primer: together with R24 used to check insertion of pK18mobsacB-fadD3. Together with extFadD3f used to check *EGO55_13795* deletion |
| 5ExtTesDf | CACTGCCAGTGGGTCATTCT | External primer: together with F24 used to check insertion of pK18mobsacB-tesD. Together with 3ExtTesDr used to check *EGO55_13685* deletion |
| 3ExtTesDr | GGGTTCTTGTCGCTCAGGAT | External primer: together with R24 used to check insertion of pK18mobsacB-tesD. Together with 3ExtTesDf used to check *EGO55_13685* deletion |
| 5ExtHSDf | AGCGCGTCTTCAGCACAGGG | External primer: together with F24 used to check insertion of pK18mobsacB-HSD. Together with 3ExtHSDr used to check *EGO55_02235*-*EGO55_02230* deletion. |
| 3ExtHSDr | GGTCGACCGATCTTCTGGAT | External primer: together with F24 used to check insertion of pK18mobsacB-HSD. Together with 5ExtHSDf used to check *EGO55_02235*-*EGO55_02230* deletion. |
| F24 | CGCCAGGGTTTTCCCAGTCACGAC | Internal primer: used to check insert cloning in pK18mobsacB and to check insertion of pK18mobsacB |
| R24 | AGCGGATAACAATTTCACACAGGA | Internal primer: used to check insert cloning in pK18mobsacB and to check insertion of pK18mobsacB |

**Table S4.** Statistical results of whole genome sequencing and mapping of all transcripts.

| **Sample** | **raw bases** | **raw reads** | **trimmed reads** | **total mapped** | **uniquely mapped (%)** |
| --- | --- | --- | --- | --- | --- |
| Pyruvate 1 | 6633517390 | 65678390 | 63360924 | 31680462 | 26789679 (84.56%) |
| Pyruvate 2 | 7687125958 | 76110158 | 73583916 | 36791958 | 30347784 (82.48%) |
| Pyruvate 3 | 9020753996 | 89314396 | 86679670 | 43339835 | 35907993 (82.85%) |
| Testosterone 1 | 3424800600 | 22832004 | 22057020 | 21921904 | 21690508 (98.34%) |
| Testosterone 2 | 3822153600 | 25481024 | 24733036 | 24577632 | 24326594 (98.36%) |
| Testosterone 3 | 3419583300 | 22797222 | 22111584 | 21978249 | 21718544 (98.22%) |

**Table S5.** Genes found in *N. tardaugens* genome homologous to genes involved in testosterone degradation in *C. testosteroni* TA441*.* Percentage of identity (ID %) and fold change (FC) increase in expression levels when *N. tardaugens* grows in testosterone are shown.

| **gene (*C. testosteroni*)** | **gene *(N. tardaugens)*** | **ID %** | **FC** |
| --- | --- | --- | --- |
| *3/17β-hsd* |  |  |  |
|  | *EGO55_02230* | 42.06 | -1.71 |
|  | *EGO55_02235* | 40.8 | 1.95 |
|  | *EGO55_02780* | 33.2 | -4.56 |
|  | *EGO55_03140* | 34.77 | -3.23 |
|  | *EGO55_03530* | 36.18 | -1.67 |
|  | *EGO55_04030* | 33.6 | -3.73 |
|  | *EGO55_04335* | 40.08 | -8.27 |
|  | *EGO55_06220* | 42.06 | -1.17 |
|  | *EGO55_06680* | 41.2 | -7.5 |
|  | *EGO55_06720* | 38.19 | -1.07 |
|  | *EGO55_06810* | 35.29 | -2.41 |
|  | *EGO55_06820* | 37.02 | -1.92 |
|  | *EGO55_06825* | 36.92 | -1.58 |
|  | *EGO55_13880* | 34.7 | -2.83 |
|  | *EGO55_14475* | 35.63 | 1.42 |
|  | *EGO55_15345* | 36.21 | -2.27 |
| *kshA* |  |  |  |
|  | *EGO55_02315* | 54.21 | 1.68 |
|  | *EGO55_02665* | 44.97 | -2.2 |
|  | *EGO55_03045* | 25.76 | -15.58 |
|  | *EGO55_04130* | 51.12 | -6.42 |
|  | *EGO55_13445* | 53.09 | 7.79 |
|  | *EGO55_16290* | 46.07 | -7.75 |
|  | *EGO55_16310* | 25.8 | -7.78 |
| *kshB* |  |  |  |
|  | *EGO55_04915* | 33 | -1.13 |
| *ksi* |  |  |  |
|  | *EGO55_02335* | 39.17 | 2.7 |
|  | *EGO55_13785* | 45.08 | 1.5 |
| *kstD (tesH)* |  |  |  |
|  | *EGO55_01175* | 41.08 | 2.35 |
|  | *EGO55_03150* | 42.28 | -2.41 |
|  | *EGO55_05950* | 33.33 | -2.14 |
|  | *EGO55_06870* | 32.44 | -1.44 |
|  | *EGO55_13510* | 44.11 | 3.53 |
| *ORF1 scdL1* |  |  |  |
|  | *EGO55_13705* | 60.34 | 2.03 |
| *ORF2 scdL2* |  |  |  |
|  | *EGO55_13710* | 54.12 | 3.08 |
| *ORF3 scdN* |  |  |  |
|  | *EGO55_13725* | 67.61 | 2.53 |
|  | *EGO55_18090* | 45.63 | -1.44 |
| *ORF4 scdK* |  |  |  |
|  | *EGO55_04015* | 42.45 | -4.2 |
|  | *EGO55_13730* | 50.7 | 3.11 |
| *ORF5 scdY* |  |  |  |
|  | *EGO55_04025* | 37.08 | -3.33 |
|  | *EGO55_04035* | 37.44 | -2.53 |
|  | *EGO55_11525* | 36.9 | -1.65 |
|  | *EGO55_13735* | 55.6 | 4.05 |
| *ORF18 scdA (fadD3)* |  |  |  |
|  | *EGO55_02270* | 29.4 | -5.32 |
|  | *EGO55_03480* | 31.74 | -2.67 |
|  | *EGO55_05005* | 30.47 | -4.95 |
|  | *EGO55_06205* | 34.33 | -2.63 |
|  | *EGO55_07035* | 32.6 | -1.51 |
|  | *EGO55_12210* | 34.19 | -2.44 |
|  | *EGO55_13795* | 49.32 | 1.24 |
|  | *EGO55_14255* | 32.8 | -14.65 |
|  | *EGO55_16325* | 31.65 | -2.07 |
| *ORF21* |  |  |  |
|  | *EGO55_06635* | 35.71 | -1.35 |
|  | *EGO55_13740* | 62.02 | 4 |
| *ORF22* |  |  |  |
|  | *EGO55_13745* | 41 | 5.4 |
| *ORF23 scdF* |  |  |  |
|  | *EGO55_05760* | 40.29 | -4.39 |
|  | *EGO55_09255* | 35.73 | 3.62 |
|  | *EGO55_13750* | 62.92 | 4.99 |
|  | *EGO55_20150* | 35.92 | 3.09 |
| *ORF25* |  |  |  |
|  | *EGO55_02680* | 40.47 | -1.56 |
| *ORF26* |  |  |  |
|  | *EGO55_03485* | 35.32 | -2.24 |
|  | *EGO55_04710* | 41.22 | -1.12 |
| *ORF27 scdE* |  |  |  |
|  | *EGO55_10165* | 36.7 | 1.07 |
|  | *EGO55_13560* | 43.21 | 1.23 |
|  | *EGO55_13760* | 50.33 | 3.6 |
| *ORF28 scdC1* |  |  |  |
|  | *EGO55_13765* | 35.28 | 3.54 |
| *ORF30 scdC2* |  |  |  |
|  | *EGO55_13770* | 51.77 | 3.1 |
|  | *EGO55_16320* | 50.25 | -7.46 |
| *ORF31 scdG* |  |  |  |
|  | *EGO55_04020* | 51.55 | -4.98 |
|  | *EGO55_13775* | 63.22 | 3.68 |
| *ORF32 scdD* |  |  |  |
|  | *EGO55_04960* | 39 | -3.33 |
|  | *EGO55_13780* | 48.99 | 3.46 |
|  | *EGO55_16330* | 36.88 | -4.13 |
| *ORF33* |  |  |  |
|  | *EGO55_05015* | 59.65 | -1.3 |
|  | *EGO55_13790* | 61.4 | 2.21 |
| *tesA2* |  |  |  |
|  | *EGO55_13440* | 44.44 | 7.15 |
|  | *EGO55_13690* | 39.26 | 1.76 |
|  | *EGO55_16230* | 38.52 | -1.1 |
|  | *EGO55_19840* | 40.31 | -1.79 |
| *tesB* |  |  |  |
|  | *EGO55_04990* | 35.4 | -6.8 |
|  | *EGO55_13700* | 41.1 | 2.62 |
| *tesD* |  |  |  |
|  | *EGO55_04995* | 30.47 | -1.54 |
|  | *EGO55_13685* | 39.62 | 1.35 |
|  | *EGO55_15045* | 28.31 | 3.74 |
| *tesE* |  |  |  |
|  | *EGO55_13680* | 60 | 1.64 |
| *tesF* |  |  |  |
|  | *EGO55_02915* | 56.17 | 1.5 |
|  | *EGO55_13675* | 55.99 | -1.04 |
| *tesG* |  |  |  |
|  | *EGO55_02910* | 56.12 | 1.75 |
|  | *EGO55_13670* | 55.82 | 3.41 |

**Table S6.** Gene expression analysis (RNA-seq) of *N. tardaugens* grown in testosterone condition compared to pyruvate. Genes located in the SD cluster (blue) and those involved in methylmalonyl-CoA pathway (green) and cofactor B12 biosynthesis pathway (orange) are highlighted

**Table S7.** Methylmalonyl-CoA degradation cluster in *N. tardaungens* NBRC 16725. Genes homologous to those described as involved in the pathway are highlighted in green.

| **gene** | **gene product** | **FC** | **strand** | **% ID** | **reference gene product (bacterial strain)** | **Reference** |
| --- | --- | --- | --- | --- | --- | --- |
| *EGO55_01990* | Protein secretion chaperonin CsaA | 15.90 | - |  |  |  |
| *EGO55_01995* | Propionyl-CoA carboxylase biotin-containing subunit (EC 6.4.1.3) | 15.33 | - | 51 | PccA (*Haloferax* *mediterranei* ATCC 33500) | [40] |
| *EGO55_02000* | Biotin synthase (EC 2.8.1.6) | 6.48 | - | 61 | BioB (*Escherichia coli* K-12 substr. MG1655) | [41] |
| *EGO55_02005* | Methylmalonyl-CoA mutase (EC 5.4.99.2) | 11.52 | - | 62 | Sbm (*Escherichia coli* str. K-12 substr. MG1655) | [42] |
| *EGO55_02010* | Enoyl-CoA hydratase (EC 4.2.1.17) | 13.35 | - |  |  |  |
| *EGO55_02015* | Methylmalonyl-CoA epimerase (EC 5.1.99.1) | 7.64 | - | 35 | MCE (*Pyrococcus horikoshii*) | [43] |
| *EGO55_02020* | Propionyl-CoA carboxylase carboxyl transferase subunit (EC 6.4.1.3) | 16.04 | - | 75 | PccB (*Rhodobacter sphaeroides*) | [44] |
| *EGO55_02025* | Transcriptional regulator, XRE family | -1.11 | + | 46 | PccR (*Rhodobacter sphaeroides*) | [44] |

**Table S8.** Steroid degradation genes in the putative testosterone degradation pathway of *N. tardaugens* (accession CP034179). Homologous genes found in the genomes of *C. testosteroni* TA441 (accession LC010134), *Sphingomonas* sp. KC8 (accession CP016306), *Pseudomonas* sp. Chol1 (accession AMSL00000000), *R. jostii* RHA1 (accession CP000431), *M. tuberculosis* H37Rv (accession AL123456.3), *M. smegmatis* mc^2^ 155 (accession CP000480), *A. estronivorus* MH-B5 (accession NZ_JRQQ00000000) and *S. denitrificans* Chol (accession LT837803) are listed and the percentage identity is shown. A cut-off value of 39 % identity was used.

| ***N. tardaugens*** | | ***C. testosteroni*** | | ***Sphingonmonas* sp.** | | ***Pseudomonas* sp.** | | ***R. jostii*** | | ***M. tuberculosis*** | | ***M. smegmatis*** | | ***A. estronivorus*** | | ***S. denitrificans*** | |
| --- | --- | --- | --- | --- | --- | --- | --- | --- | --- | --- | --- | --- | --- | --- | --- | --- | --- |
| **gene** | **gene product** | **gene** | **% ID** | **gene** | **% ID** | **gene** | **% ID** | **gene** | **% ID** | **gene** | **% ID** | **gene** | **% ID** | **gene** | **% ID** | **gene** | **% ID** |
| *EGO55_13670* | 4-hydroxy-2-oxovalerate aldolase | *tesG* | 55.82 | *KC8_03550* | 93.37 | *C211_08579* | 74.77 | *RHA1_ro08083* | 68.96 | *Rv3534c* | 51.37 | *MSMEG_4150* | 61.68 | *MB02_RS11275* | 92.73 | *-* | - |
| *EGO55_13675* | acetaldehyde dehydrogenase (acetylating) | *tesF* | 55.99 | *KC8_03555* | 86.67 | *C211_08574* | 63.91 | *RHA1_ro00516* | 58.75 | *Rv3535c* | 55.67 | *MSMEG_4149* | 59.80 | *MB02_RS17275* | 93.94 | *-* | - |
| *EGO55_13680* | 2-hydroxypenta-2,4-dienoate hydratase | *tesE* | 60.00 | *KC8_03560* | 78.28 | *C211_11267* | 58.87 | *RHA1_ro04533* | 43.93 | *Rv3536c* | 42.74 | *MSMEG_5940* | 43.59 | *MB02_RS16020* | 91.01 | *-* | - |
| *EGO55_13685* | alpha/beta fold hydrolase | *tesD* | 39.62 | *KC8_19645* | 69.00 | *C211_11432* | 42.08 | *-* | - | *-* | - | *MSMEG_2913* | 40.74 | *MB02_RS16015* | 85.24 | *-* | - |
| *EGO55_13690* | hydroxylase | *tesA2* | 39.26 | *KC8_03565* | 71.96 | *C211_11297* | 50.13 | *-* | - | *-* | - | *MSMEG_3911* | 40.00 | *MB02_RS16010* | 88.12 | *-* | - |
| *EGO55_13695* | SDR family oxidoreductase | *BAP91391.1_62* | 46.61 | *KC8_01160* | 52.65 | *C211_11357* | 48.62 | *RHA1_ro04653* | 49.39 | *Rv3549c* | 49.19 | *MSMEG_6000* | 49.21 | *MB02_RS16005* | 83.46 | *SDENCHOL_10469* | 50.78 |
| *EGO55_13700* | extradiol dioxygenase | *tesB* | 41.10 | *KC8_01090* | 40.60 | *C211_11492* | 50.84 | *-* | - | *-* | - | *-* | - | *MB02_RS16000* | 81.13 | *-* | - |
| *EGO55_13705* | CoA transferase subunit alfa | *ORF1* | 60.34 | *KC8_01085* | 68.15 | *C211_11497* | 56.16 | *RHA1_ro04651* | 49.82 | *Rv3551* | 46.32 | *MSMEG_6002* | 49.65 | *MB02_RS15995* | 90.03 | *SDENCHOL_10317* | 57.19 |
| *EGO55_13710* | CoA transferase subunit beta | *ORF2* | 54.12 | *KC8_01080* | 78.46 | *C211_11502* | 57.69 | *RHA1_ro04650* | 40.86 | *Rv3552* | 42.75 | *MSMEG_6003* | 40.30 | *MB02_RS15990* | 90.87 | *SDENCHOL_10318* | 60.98 |
| *EGO55_13715* | benzoylsuccinyl-CoA thiolase | *-* | - | *KC8_01075* | 75.35 | *-* | - | *-* | - | *Rv1628c* | 46.04 | *MSMEG_3843* | 45.65 | *MB02_RS15985* | 91.61 | *SDENCHOL_10319* | 58.87 |
| *EGO55_13720* | lipid-transfer protein | *-* | - | *KC8_01070* | 83.38 | *-* | - | *-* | - | *Rv1627c* | 66.75 | *MSMEG_3844* | 65.99 | *MB02_RS15980* | 94.66 | *SDENCHOL_10320* | 85.97 |
| *EGO55_13725* | enoyl-CoA hydratase | *ORF3* | 67.61 | *KC8_09445* | 82.81 | *C211_11507* | 67.01 | *RHA1_ro04594* | 59.55 | *-* | - | *MSMEG_4846* | 43.25 | *MB02_RS15975* | 92.43 | *-* | - |
| *EGO55_13730* | nitronate monooxygenase | *ORF4* | 50.70 | *KC8_01065* | 72.36 | *C211_11512* | 49.29 | *RHA1_ro04649* | 46.31 | *Rv3553* | 45.17 | *MSMEG_6004* | 45.69 | *MB02_RS15970* | 92.70 | *SDENCHOL_10321* | 49.86 |
| *EGO55_13735* | enoyl-CoA hydratase family protein | *ORF5* | 55.60 | *KC8_01060* | 73.60 | *-* | - | *RHA1_ro04652* | 47.01 | *Rv3550* | 43.90 | *MSMEG_6001* | 47.01 | *MB02_RS15965* | 93.98 | *SDENCHOL_10322* | 52.48 |
| *EGO55_13740* | acyl-CoA dehydrogenase | *ORF21* | 62.02 | *KC8_01055* | 64.42 | *C211_11527* | 60.82 | *RHA1_ro04593* | 52.81 | *Rv3562* | 50.65 | *MSMEG_6014* | 50.52 | *MB02_RS15960* | 91.69 | *SDENCHOL_10323* | 65.13 |
| *EGO55_13745* | acyl-CoA dehydrogenase | *ORF22* | 41.00 | *KC8_01050* | 41.95 | *C211_11532* | 42.00 | *-* | - | *-* | - | *-* | - | *MB02_RS15955* | 88.34 | *SDENCHOL_10324* | 44.16 |
| *EGO55_13750* | acetyl-CoA C-acetyltransferase | *ORF23* | 62.92 | *KC8_01045* | 69.27 | *C211_11537* | 63.90 | *RHA1_ro04599* | 63.54 | *Rv3556c* | 59.54 | *MSMEG_6008* | 61.72 | *MB02_RS15950* | 84.64 | *SDENCHOL_10325* | 62.34 |
| *EGO55_13755* | nuclear transport factor 2 family protein | *-* | - | *KC8_18165* | 63.89 | *-* | - | *-* | - | *-* | - | *-* | - | *MB02_RS11790* | 40.85 | *-* | - |
| *EGO55_13760* | SDR family NAD(P)-dependent oxidoreductase | *ORF27* | 50.33 | *KC8_01035* | 81.67 | *C211_11542* | 50.33 | *RHA1_ro04654* | 55.12 | *Rv3548c* | 54.61 | *MSMEG_5999* | 58.14 | *MB02_RS15945* | 91.33 | *SDENCHOL_10772* | 54.97 |
| *EGO55_13765* | acyl-CoA dehydrogenase | *ORF28* | 35.28 | *KC8_01030* | 41.71 | *-* | - | *-* | - | *-* | - | *MSMEG_4875* | 40.77 | *MB02_RS15940* | 84.33 | *-* | - |
| *EGO55_13770* | acyl-CoA dehydrogenase | *ORF30* | 51.77 | *KC8_03525* | 64.47 | *C211_11342* | 58.38 | *RHA1_ro04596* | 55.22 | *Rv3560c* | 53.87 | *MSMEG_6012* | 52.91 | *MB02_RS15935* | 87.24 | *SDENCHOL_10770* | 57.43 |
| *EGO55_13775* | SDR family oxidoreductase | *ORF31* | 63.22 | *KC8_01025* | 79.62 | *C211_11547* | 64.37 | *RHA1_ro04597* | 60.54 | *Rv3559c* | 61.15 | *MSMEG_6011* | 60.92 | *MB02_RS15930* | 90.77 | *SDENCHOL_10769* | 63.18 |
| *EGO55_13780* | MaoC family dehydratase | *ORF32* | 48.99 | *KC8_01020* | 68.67 | *C211_11552* | 59.18 | *RHA1_ro02723* | 56.38 | *Rv0130* | 48.63 | *MSMEG_2201* | 51.02 | *MB02_RS15925* | 92.00 | *SDENCHOL_10768* | 54.73 |
| *EGO55_13785* | steroid Delta-isomerase | *ksi* | 45.08 | *KC8_01015* | 64.23 | *-* | - | *-* | - | *-* | - | *-* | - | *MB02_RS15920* | 89.52 | *SDENCHOL_10767* | 43.90 |
| *EGO55_13790* | acetyl-CoA C-acyltransferase | *ORF33* | 61.40 | *KC8_01010* | 73.75 | *C211_11557* | 59.65 | *-* | - | *-* | - | *-* | - | *MB02_RS15915* | 92.25 | *SDENCHOL_10525* | 61.65 |
| *EGO55_13795* | fatty-acid-CoA ligase | *ORF18* | 49.32 | *KC8_01165* | 48.71 | *C211_11322* | 48.17 | *RHA1_ro04595* | 46.14 | *Rv3561* | 47.84 | *MSMEG_4772* | 45.56 | *MB02_RS15910* | 79.61 | *SDENCHOL_10766* | 49.32 |

**Figure S1.** 12.5% SDS-polyacrylamide gel electrophoresis of the overproduction of Hsd70-Hsd60, Hsd60 and Hsd70 proteins in the soluble fraction of the crude extract of *E. coli* BL21(DE3) strains. 25 µg of total protein of each sample where loaded.


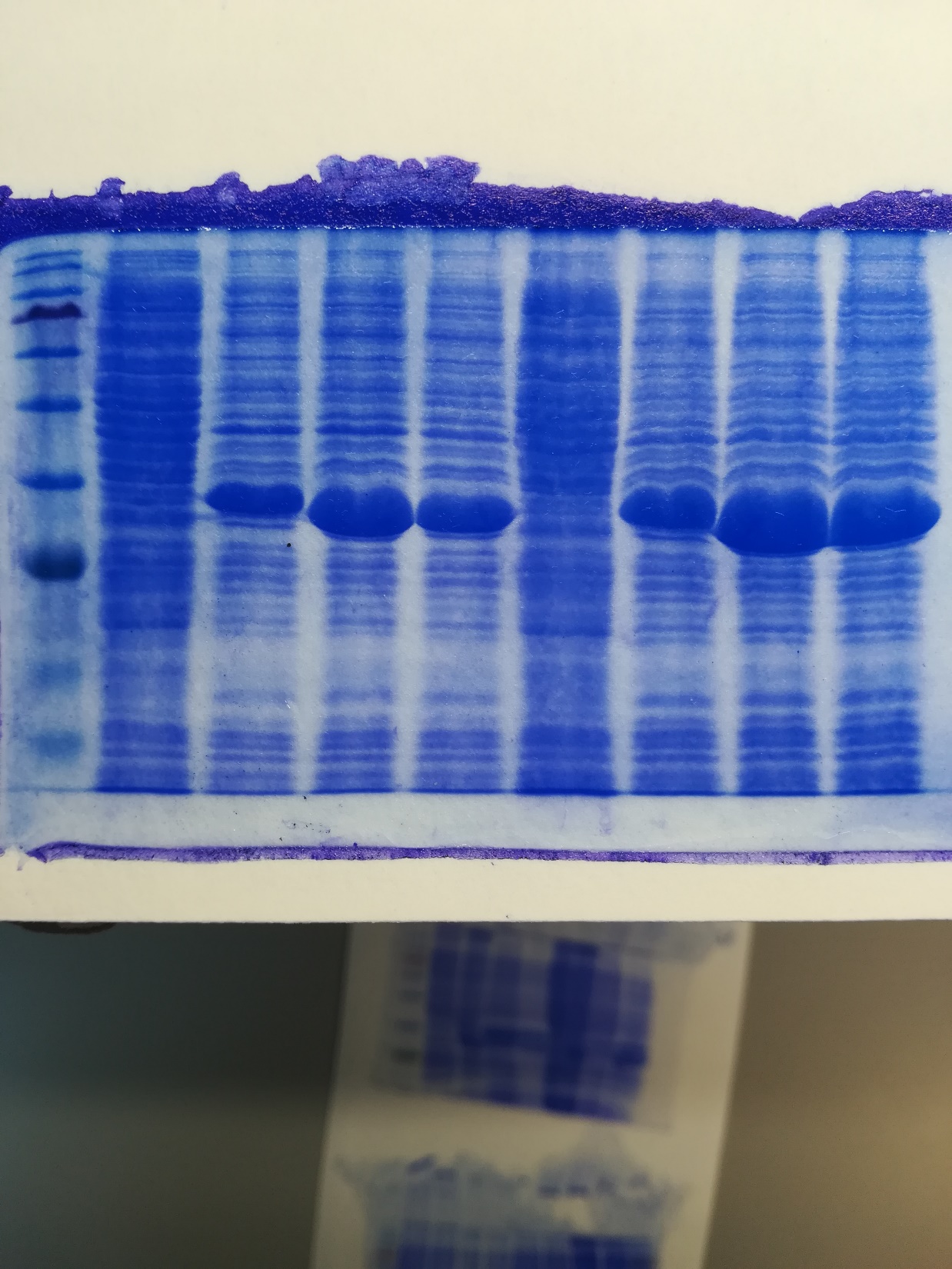


pET29Hsd60-Hsd70

pET29Hsd60

pET-29a(+)

pET29Hsd70

35 kDa

28 kDa

17 kDa

10 kDa

63 kDa

48 kDa

180 kDa

75 kDa

**Figure S2.** Bacterial growth (OD_600_) of *N. tardaugens* NBRC 16725 (red), *N. tardaugens* Δ*fadD3* (yellow), *N. tardaugens* Δ*tesD* (purple) and *N. tardaugens* Δ*hsd* (green) strains when cultured in M63 minimal medium containing 1.89 mM TES.

**Figure S3.** Protein alignment of the 3α,20β-HSD from *Streptomycens* *hydrogenans* with HSD60 and HSD70. The key amino acids of the active site are in red. The variable sequence is shown in a red box. This sequence is involved in substrate binding in the 3α,20β-HSD from *S. hydrogenans*.
